# Biocompatible and Biodegradable 3D Printing from Bioplastics: A Review

**DOI:** 10.3390/polym15102355

**Published:** 2023-05-18

**Authors:** Maurine Naliaka Andanje, James Wamai Mwangi, Bruno Roberts Mose, Sandro Carrara

**Affiliations:** 1 Department of Mechatronic Engineering, Jomo Kenyatta University of Agriculture and Technology (JKUAT), Nairobi 00200, Kenya; 2 Department of Mechanical Engineering, Jomo Kenyatta University of Agriculture and Technology (JKUAT), Nairobi 00200, Kenya; 3 Institute of Electrical and Micro Engineering, École Polytechnique Fédérale de Lausanne (EPFL), 1015 Lausanne, Switzerland

**Keywords:** bioplastics, biocomposites, biopolymers, 3D printing, fused filament fabrication

## Abstract

There has been a lot of interest in developing and producing biodegradable polymers to address the current environmental problem caused by the continued usage of synthetic polymers derived from petroleum products. Bioplastics have been identified as a possible alternative to the use of conventional plastics since they are biodegradable and/or derived from renewable resources. Additive manufacturing, also referred to as 3D printing, is a field of growing interest and can contribute towards a sustainable and circular economy. The manufacturing technology also provides a wide material selection with design flexibility increasing its usage in the manufacture of parts from bioplastics. With this material flexibility, efforts have been directed towards developing 3D printing filaments from bioplastics such as Poly (lactic acid) to substitute the common fossil- based conventional plastic filaments such as Acrylonitrile butadiene styrene. Plant biomass is now utilized in the development of biocomposite materials. A lot of literature presents work done toward improving the biodegradability of printing filaments. However, additive manufacture of biocomposites from plant biomass is faced with printing challenges such as warping, low agglomeration between layers and poor mechanical properties of the printed parts. The aim of this paper is to review the technology of 3D printing using bioplastics, study the materials that have been utilized in this technology and how challenges of working with biocomposites in additive manufacture have been addressed.

## 1. Introduction

Plastic materials are key in today’s world, finding applications in a variety of industries, including consumer products, automotive, agriculture, aerospace, electronics and health care. While plastics continue to be popular due to their durability, adaptability, and cost-effectiveness, the continual depletion of petroleum resources to produce plastic materials and the environmental pollution caused by plastic disposal has aroused global concern. Efforts have been made globally to deal with the increasing amounts of plastic waste and to develop sustainable industrial materials [1,2,3,4].

Bioplastics are a promising complement to conventional plastics since they are biocompatible and have the potential for biodegradability [5]. Bioplastics are defined as plastics that are either derived from renewable natural resources (bioderived) and/or biodegradable [6,7,8,9,10]. Such natural resources include starch, chitin, protein and cellulose [11]. Biodegradable bioplastics provide disposal methods that reduce the quantity of plastic waste that ends up in the environment. Bioderived bioplastics, on the other hand, allow for a significant reduction in carbon footprint at the resource extraction stage. Biomass used for developing bioplastics is currently derived from starch constituents or cellulose.

The chemical structure of a bioplastic determines whether it is biodegradable; for example, a 100% bioderived bioplastic might not be biodegradable [12].

Despite the fact that bioplastics have been studied for almost a century, their widespread manufacturing is still in its early stages. The European Bioplastics Association reported a global bioplastic production of 2.11 MT in 2019, accounting for barely 0.6% of overall plastics production. This is expected to reach 2.89 MT by 2025 [10,13]. The major reasons for the slow adoption of bioplastics are their higher production costs and lower mechanical properties as compared to conventional plastics. Also, when plants are produced for bioplastics, there are additional environmental considerations, such as food competition and recycling problems [6,12,14]. Nonetheless, because the world urgently requires a viable substitute for petroleum-based plastics, the bioplastics market is likely to develop significantly in the coming years, outpacing the petrochemical plastics market. Efforts have been made towards improving the mechanical properties of bioplastic materials to make them compete favorably with conventional plastics.

Many biocomposites now offer improved material properties; higher breathability, increased material strength [15], reduced thickness and improved optical properties, which all contribute to better material performance. These materials are naturally derived from proteins, lipids, aliphatic polyesters, or polysaccharides (obtained from agricultural products, livestock and fish farming) and are seen to be potential substitutes for conventional plastics due to their biocompatibility, safety and rate of biodegradation [16,17]. These polymers are quickly broken down by natural microorganisms in the presence of the proper temperature, moisture and oxygen without causing any environmental harm. Polysaccharides contain starch, cellulose, gum, alginate, chitosan, carrageenan, pectin, pullulan, or their derivatives [18]. Starch constituents commonly used to produce bioplastic materials put pressure on food security. An alternative way to deal with this challenge is to make use of plant waste materials, such as agricultural by-products, in combination with recycled plastics to produce the bioplastics [19,20,21,22].

Agricultural residues have recently emerged as a possible feedstock material in additive manufacturing processes [23,24]. Improved printing directionality [25,26], enhanced mechanical properties [20,27], and reduced warping [19,22] have been reported with the use of agricultural residues. Additive manufacturing is a group of manufacturing techniques that has brought a paradigm shift in manufacturing in which objects are produced by materials joining layer upon layer. Recent developments in these techniques have made it possible to design and produce almost any product from a wide variety of materials with much flexibility, which was not possible with previous techniques. This group of techniques will facilitate the shift from the use of fossil-based materials to biocomposites as feedstocks, therefore, providing an opportunity to realize a more sustainable green economy [28,29,30].

This paper reviews the different bioplastics developed from natural resources that can be used as feedstock in different additive manufacturing (AM) technologies. The objectives are to find alternative ways to switch from plastics made from fossil fuels to biopolymers obtained from natural sources for AM, as well as to encourage a shift in the design of products and their production towards sustainability. We first present the different bioplastics as well as bioplastics with biocompatibility that are available commercially, followed by methodologies for assessing their biodegradability. A brief description of some commercially available additive manufacturing technologies suitable for biocomposites is reviewed, and research work on additive manufacturing using biocomposites reinforced with biobased fillers is presented. Lastly, the key challenges and opportunities for AM using bioplastics obtained from natural sources have been highlighted.

## 2. Bioplastics with Biodegradability

Thermoplastics such as polyethylene terephthalate (PET), polyethylene (PE), polypropylene (PP) and polystyrene (PS) currently account for 60% of the total plastic demand [31]. While these plastics are generally made from petrochemicals, there is an increasing demand for plastics derived from renewable resources as alternatives to petrochemically-derived plastics. These new alternative plastics are known as bioplastics, which are biobased, biodegradable or both [9,32].

Three types of bioplastics are distinguished:

- Biodegradable bioplastics made from biobased materials. Examples include poly (lactic acid) (PLA), polyhydroxyalkanoates (PHAs), thermoplastic starch (TPS), and poly (butylene succinate) (PBS) [33];
- Biodegradable bioplastics made from petrochemical resources. Examples include Poly (butylene adipate terephthalate) (PBAT) and polycaprolactone (PCL) [34];
- Non-biodegradable or partially biodegradable bioplastics from biobased monomers and bioderived technical polymers. Examples are bioPE, bioPET, bioPP, and poly-trimethylene terephthalate (bioPTT) [35].

**Figure 1:**
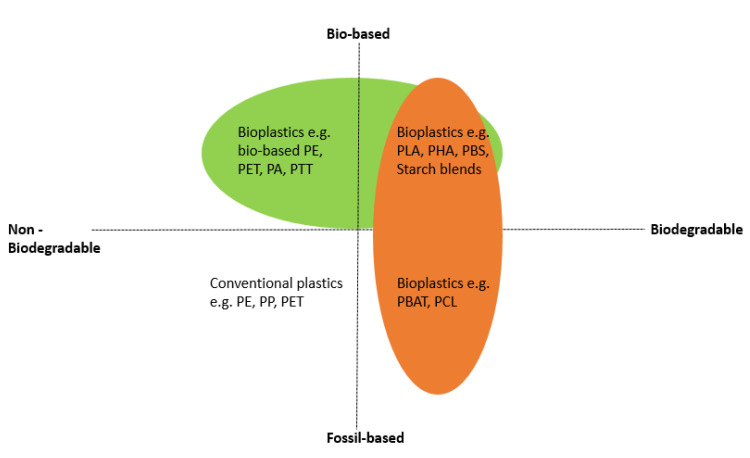
Classification of plastics according to their origin and biodegradability (Reprinted with permission from Ref. [9]) (Copyright 2021 Elsevier B.V.).

Figure 1 shows the classification of plastics and bioplastics according to their origin and biodegradability.

Biodegradable biobased plastics can be recycled or incinerated in the same way as conventional plastics; however, they are not extensively recycled since they are considered pollutants in the present recycling system [36]. They are primarily designed to degrade under precise conditions, most typically in a controlled setting in industrial composting plants [37]. Unless they are designed to biodegrade in a particular environment, such as soil or water, they will not degrade or will degrade very slowly in these conditions [11].

Table 1 shows some common bioplastics compositions, their properties and areas of application. The porosity, size, and carbon content of bioplastics alter as they degrade, affecting their ability to contain and retain water [38].

### 2.1. Methodologies for Assessing the Biodegradability of Bioplastics

The rate of biodegradation of bioplastics varies based on factors such as external environmental variables, intrinsic physicochemical features of the biopolymer, or filler characteristics in blends/composites [51,52,53]. Biodegradation can be classified as biological degradation, whereby microorganisms produce enzymes that are responsible for degradation. It can be classified as chemical degradation, whereby agents such as water, oxygen, acids, alkalines and solvents are used for the breakdown of the biopolymer. Other types of biodegradation are physical degradation, atmospheric degradation and hydrolytic degradation, a unique form of chemical degradation [54].

**Figure 2:**
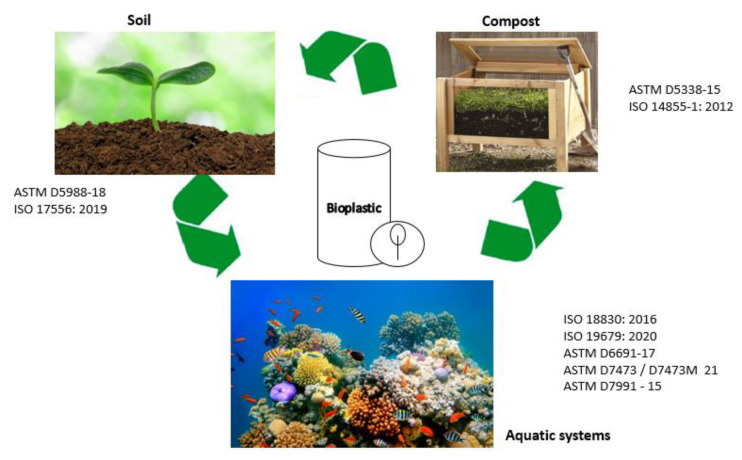
International standards for evaluating the biodegradability of bioplastics (Reprinted with permission from Ref. [54] Copyright 2022, Polymers).

The existing methodologies for assessing the biodegradability of bioplastics can be classified into three categories based on where the bioplastic is disposed of [34,54]: (i) soils, (ii) compost, and (iii) aquatic systems. Figure 2 shows these three categories for evaluating biodegradability and their respective standards.

### 2.2. Biodegradation in Soil

The two most common standard methodologies for testing the biodegradation of plastics in soil are ASTM D5988-18, “standardized test to find aerobic plastic biodegradation in soil” and ISO 17556:2019, “determining the absolute aerobic biodegradability of plastic materials in soil by measuring carbon dioxide production or oxygen demand in a respirometer” [55,56]. ASTM D5988-18 estimates the quantity of carbon dioxide produced by microorganisms as a function of exposure duration, hence assessing the degree of biodegradability with comparison to a reference material. Similarly, the ISO 17556:2019 method controls oxygen intake or carbon dioxide production to produce the best rate of plastic biodegradation in test soil. In both cases, the bioplastic sample is essentially buried in closed containers containing soil that has been previously prepared. The containers are then exposed to a temperature range that promotes mesophilic microbe development, together with optimal moisture and oxygen conditions. Table 2 shows the different methodologies to assess biodegradability in soil for some developed biopolymers.

The most fundamental and extensively used biodegradability index is mass loss. This methodology entails the measurement of samples collected at various periods and drying them until they reach a constant weight [57,58,59]. The gel permeation chromatography used to determine molecular weight is another technique for detecting mass loss [60].

Scanning electron microscopy (SEM) morphology analysis can be used to analyze biodegradability in soil. This technique is for determining the influence of water intrusion in biopolymers, which is known as surface erosion. Furthermore, using this technique, the identification and evaluation of microorganisms growing on the surface can be studied during biodegradation. This methodology was used by Jana et al. [61], whereby SEM was used to observe the breakdown of PHA and PBS samples, as well as the formation of biofilms on material surfaces. On the contrary, microorganisms did not cover two slowly degradable polymers, that is, PBAT/PLA and ICL- PN. Their findings matched the CO_2_ measurements and, more importantly, the microbiological assessment of the biodegradation process.

**Table 2:** Methodologies to assess biodegradability in soil.

Biopolymer	Soil Conditions	Methodology	Ref.
PBAT/Nanocellulose	Lab-controlled conditions	ASTM D5988	[62]
Cellulose-based	Lab-controlled conditions	ASTM D5988	[63]
PHBV/Olive Pomace	Lab-controlled conditions	ASTM D5988	[64]
Chitosan/Corn cob	Lab-controlled conditions	ASTM D5988	[65]
Polyurethane (PU)/Starch	Lab-controlled conditions	ASTM D5988 visual analysis, morphological and chemical characterization	[66]
PLA/Glycerol	Lab-controlled conditions	ISO 17556	[67]
Starch/Nanocellulose	Lab-controlled conditions	ISO 17556	[68]
PBS, PHA, PBAT/PLA	Lab-controlled conditions	Microrganismo characterization (ISO 17556)	[61]
PBAT, starch, and additives mixture	Lab-controlled conditions	Ecotoxicological analysis (ISO 17556)	[61]
Compounds of PHB and natural fillers	Lab-controlled conditions	Mass loss	[59]
Starch/Nanocellulose	Outdoor conditions	Mass Loss	[57]
PVA/starch	Lab-controlled conditions	Mass loss, visual analysis, biofilm area, and soil characterization	[58]
PLA, PBS, PHB and PCL	Lab-controlled and outdoor conditions	Mass loss, microbial characterization and mechanical properties	[69]
Starch-based	Lab-controlled conditions	Mass loss, mechanical properties and morphological analysis	[70]
PBS/Sugarcane Fibre	Lab-controlled conditions	Mass loss, morphological analysis, and thermal characterization	[71]
PHA	Outdoor conditions	Mass loss, chemical and morphological analysis	[72]
PLA and PLA/starch	Outdoor conditions	Mass loss, thermal characterization, morphological and chemical analysis	[60]
Mixtures of PVA/starch with natural fillers	Lab-controlled conditions	Mass loss, soil characterization, morphological and chemical analysis	[73]

Mechanical characterization has been used to analyze biodegradability in soil. This was used by Ibrahim et al. [70], who compared the mechanical characteristics of starch biocomposites that were reinforced with different ratios of lignocellulosic fibers to the biocomposites’ biodegradability. The tensile strength and modulus of elasticity of the biocomposites were found to have decreased by more than 50% within the first week and subsequently gradually worsened until the experiment’s conclusion. Sabapathy et al. [72] used chemical analysis to test for biodegradation of PHA. Using Fourier transform infrared spectroscopy (FTIR), the researchers looked at the shift and intensity of specific infrared peaks to calculate the degree of degradation.

### 2.3. Biodegradation in Compost

Composting is an aerobic form of solid waste treatment where biodegradable materials are organically broken down into humus. In the presence of microorganisms and under controlled conditions, humus is a beneficial nutrient source for increasing soil productivity and agricultural yield. This method is very helpful in resolving the disposal issue and reducing greenhouse gas emissions since compost is a healthy organic substrate that can be returned to the ecosystem [74,75].

Most methodologies of biodegradation of biopolymers in aerobic composting conditions have more standardizations than in soils due to the fact that these tests are typically carried out in laboratories. The common standards are shown in Table 3. The most often used standards are ISO 14855-1:2012 [76] and ASTM D5338 -15 [77]. Other standards used for testing biopolymer biodegradability in compost include the ASTM D6400-21 [78], ISO 17556 [56], ISO 20200 [79], and ISO 17088:2021 [80].

Kalita et al. [74] investigated the aerobic biodegradation of PLA biocomposite films, which were made by modifying PLA with the addition of fillers like chitosan and gum. Biodegradation was studied using a variety of analytical approaches, including differential scanning calorimetry (DSC), molecular weight analysis, microbial colony count and contact angle analysis. The findings indicated that modified PLA films are biodegradable, as well as the applicability of the methodologies for studying biopolymer biodegradation.

### 2.4. Biodegradation in Aquatic Systems

The catastrophic pollution situation that is currently afflicting aquatic systems is primarily due to plastic waste. This pollutant begins its flow in wastewater treatment effluents, which then make their way to fresh inland waters such as lakes and rivers, before continuing on to the oceans, where it settles and continues to break down into micro- and nano-plastics. The growth, behavior, development, reproduction, and life-span of marine and freshwater species may be impacted by the created debris [86,87]. Table 4 highlights the most common biodegradability standards in aquatic systems that have been developed by ISO and ASTM.

## 3. Bioplastics with Biocompatibility

Biocompatible polymers are gaining popularity, especially in the biomedical field. The word “biocompatibility” refers to a polymer’s appropriateness for exposure to the human body and bodily fluids. These are used to assess, treat, augment, or replace any body tissue, organ, or function. A biocompatible polymer increases bodily functions without disrupting normal function or causing allergies or other negative side effects [88].

Biodegradable aliphatic polymers such as PLA, PCL, Poly lactic-co-glycolic acid (PLGA), polyhydroxyalkanoate (PHA), as well as related copolymers, are now used in biomedical applications in the human body and hence considered to be biocompatible [89]. The backbones of many biodegradable polymers contain hydrolyzable connections like carbonate, anhydride, urea, urethane, amide, esters and orthoester. Aliphatic polymers with ester linkages have excellent biocompatibility and a wide range of physical, chemical, and biological characteristics. Due to their biocompatibility, PLA and PHA are the most commonly used aliphatic polymers in biomedical applications [90,91].

Long-term treatments and performances require an appropriate and consistent relationship between the living environment and the material. The situation, however, is very different due to bioresorbable and biodegradable polymers, where there is a great deal of complexity due to the by-products of implant degradation and resorption that could interact powerfully with biological systems. Therefore, before a polymer is selected for medical implants, its biodegradability and bioresorbability must be evaluated. The release of acidic substances from the body implant during degradation is a major factor for body inflammation. The location of the implantation is another factor that affects inflammation response in the host body [92]. The following subsection gives a brief description of biocompatible bioplastics stating their main application areas.

### 3.1. Poly (Lactic Acid)

Poly (lactic acid) (PLA) is the most extensively utilized biopolymer in medical applications due to its biocompatibility and biodissolvability in the human body via the hydrolysis of the ester backbone, which results in non-harmful and non-toxic chemicals. Gregor et al. [93] designed PLA scaffolds for bone tissue replacement using the 3D printing technique. According to the cell proliferation experiments and mechanical testing of constructed scaffold samples, it was determined that the recommended porosity of the scaffold for bone tissue replacement of approximately 90% was unnecessary. Qingwei et al. [94] successfully fabricated PLA/octa-decyl amine composites that were functionalized with nano-diamond (ND-ODA) for tissue engineering applications. They also showed how PLA and its copolymers, such as PLA-p-dioxanone-polyethylene glycol (PLA-p-DPEG) copolymer and PLA polyethylene glycol (PLA-PEG) copolymer, can be used as carriers for bone morphogenetic proteins.

Other biocompatibility applications of PLA include [95]: wound management, delivery systems, orthopedic implants, dentistry, and skin and tendon regeneration.

### 3.2. Poly Lactic-Co-Glycolic Acid

Poly lactic-co-glycolic acid (PLGA) has received approval from both the European Medicine Agency and the American FDA (US Food and Drug Administration) as one of the best synthetic biodegradable polymers used in the biomedical sector. The material has attracted a lot of interest as a primary material for medical applications because of its biocompatibility and biodegradation rates, which depend on the molecular weight and copolymer ratio of the polymer.

PLGA is a crystalline, hydrophilic polymer with a relatively fast degradation rate compared to other biodegradable polymers. For the mixing of bone replacement structures, PLGA co-polymers are highly preferred over their constituent homo-polymers since maintaining high-grade control over their degrading qualities is recommended by adjusting the quantities of their monomers.

PLGA finds application in bone tissue engineering. Sanghwa et al. [96], for instance, fabricated multi-layered 3D scaffolds by stacking microfibrous PLGA meshes alternately with micro/nano mixed PLGA and collagen fibrous meshes with and without hydroxyapatite nanorods (nHA). In comparison to pristine microfibrous PLGA and micro/nano mixed fibrous PLGA and Col scaffolds, the micro/nano fibrous PLGA-Col-HA scaffolds were found to be highly bioactive. PLGA also finds application in injectable microspheres [97] and in dentistry [98].

### 3.3. Poly (ε-Caprolactone)

Poly (ε-caprolactone) (PCL) is semi-crystalline and hydrophobic, with crystallinity decreasing as molecular weight increases. PCL’s features, such as its high solubility, low melting point (59–64 °C), and exceptional blending compatibility, have prompted much re-search into its potential biomedical applications [99]. PCL has several features that make it suited for controlled drug delivery: high permeability for a variety of medicines, strong biocompatibility, and the capacity to entirely excrete from the body once bio-resorbed. Since PCL biodegrades at a slower rate than other polymers, it is best suited for long-term drug delivery systems that can last up to a year. PCL can also create compatible blends by incorporating other polymers, which can affect degradation kinetics and make it easier to adjust to meet desired drug release profiles [90].

Because of its low melting point and remarkable rheological and mechanical qualities, PCL has drawn significant interest as a biomaterial in the fields of cardiovascular and bone tissue engineering. It lends itself nicely to the construction of scaffolds. Additionally, it is a highly versatile bioresorbable polymer that, due to its excellent rheological properties, may be used in almost any polymer processing technology to create a wide range of scaffolds [100].

## 4. Additive Manufacturing of Biocomposites

A biocomposite is a composite material made up of a resin matrix that is reinforced with natural fiber. These materials frequently replicate the structure of the biological components employed in the process while maintaining the matrix’s strength and always ensuring biocompatibility. Polymers made from sustainable and non- renewable resources make up the matrix phase. The matrix protects the fibers from environmental degradation and mechanical damage while also holding them together and transferring stresses. Additionally, biofibers are also the main constituents of biocomposites, which are made from biological sources such as crop fibers (cotton, hemp or flax), recycled waste paper, wood, byproducts from crop processing, fibers from animals (flax, silk, hemp, feathers, kenaf, and wool) and fiber from regenerated cellulose (viscose/rayon) [101,102,103]. Cellulose, a common reinforcing material for biocomposites, is reviewed below.

### 4.1. Cellulose

Cellulose, the primary structural element of plant cell walls, is the most prevalent renewable biopolymer. The application of cellulose in composite materials has attracted a lot of attention due to its exceptional biodegradability, combustibility, non-abrasiveness, affordability, and low-density properties [103].

#### 4.1.1. Natural Fibers Reinforced Composites from Cellulose

As a green and environmentally friendly material, natural fiber-reinforced composites (NFRCs) made of cellulosic resources have gained popularity. NFRCs, also known as “green composites,” are made by incorporating several kinds of natural fibers (NFs) into biodegradable resins and starch as a polymer matrix to enhance the mechanical properties of green composites. Compared to traditional composite materials, they are simple to manufacture and have a small impact on the environment [104,105,106,107]. Just like synthetic fibers, these fibers are made using extrusion processes [108], resin transfer molding [109], manual lay-up [110], compression molding [111] and injection molding processes [112].

NFRCs can be made from cellulose materials, which are more affordable and sustainable than traditional composites. However, these materials have a number of significant shortcomings that negatively affect their mechanical qualities, such as increased moisture absorption, poor wetting characteristics, incompatibility with the matrix, and low thermal resistance [113,114]. Due to these shortcomings, green composites are limited in their application. Additionally, the inconsistent nature of the NFs makes it difficult to fabricate composites, which further restricts their industrial application [115,116].

#### 4.1.2. Nanocellulose

Natural cellulose fibers from cotton linters, plant cell walls, or microorganisms are converted into nanocellulose using mechanical, enzymatic, or chemical methods. The fibers have a diameter of less than 100 nm and a few micrometers in length [117,118]. The three main types of nanocellulose include nano fibrillated cellulose (NFC) [119], cellulose nanocrystals (CNC) [120], and bacterial nanocellulose (BNC) [121]. Table 5 shows a comparison of the properties of the main types of nanocellulose.

Since the effectiveness of nanocellulose-based materials depends on the achieved accuracy in nanocellulose dimensions, the dimensions of nanocellulose extracted from various plants and natural fibers are crucial. High modulus of elasticity (110–220 GPa), high tensile strength (7.5–7.7 GPa), tailored aspect ratios, high degree of polymerization, high surface functionalization, flexible crystallinity, high chemical resistance and high specific surface area are just a few of nanocellulose’s exceptional qualities. As a result of these distinctive physicochemical features, nanocellulosic sustainable materials are becoming more important in the automotive, aerospace, energy, packaging and other multidisciplinary industries [122,123].

By imitating the composition and structure of the plant cell walls, additive manufacturing technologies could provide novel approaches to the design and production of new materials. This article reviews three categories of AM technologies frequently used with biobased materials. These include [25]:

- Material extrusion methods such as fused filament fabrication (FFF), direct ink writing (DIW), and micro-extrusion 3D bioprinting;
- Inkjet 3D printing;
- 3D spinning.

Table 6 shows some typical cellulose-reinforced plastic filaments developed for use in FFF.

### 4.2. AM Technologies Used with Biobased Materials

#### 4.2.1. Material Extrusion Methods

**Figure 3:**
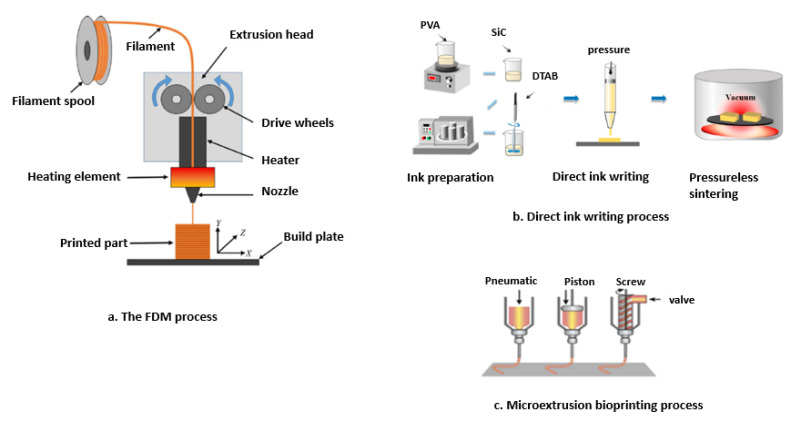
Material extrusion methods: (**a**) The FDM process where material is extruded through a heated nozzle (Adapted with permission from Ref. [134] Copyright 2023, Scientific Research Publishing Inc.), (**b**) Direct ink writing where liquid-phase ”ink” is deposited from tiny nozzles at controlled flow rates and placed along digitally determined routes (Adapted with permission from Ref. [135] Copyright 2023, MDPI, Basel, Switzerland), (**c**) Microextrusion bioprinting system [136] where 3D structures are created by layer-by-layer precisely arranging biological materials, chemical materials and living cells with positional control of functional components (Adapted with permission from Ref. [131] Copyright 2014, Nature Biotechnology).

This is one of the most widely used AM technologies, which involves dispensing extrudable material via a nozzle and depositing it layer by layer. The availability of a wide range of materials for this method is what has contributed to its popularity. This category includes the processes of fused deposition modeling (FDM)/fused filament fabrication (FFF) [128,129], direct ink writing (DIW) [130], and microextrusion 3D bioprinting [131]. The schematics for these processes are shown in Figure 3.

Reports indicate that biobased materials, such as cellulose nanoparticles and lignocellulosic material, are used in extrusion-based 3D printing techniques. The production of filaments with smaller diameters used in extrusion- based 3D printing is still extremely difficult because of die swell, which occurs when viscous liquefied materials are extruded via a nozzle with a small diameter. The quality of 3D-printed parts from biobased materials depends on the printability of these materials [25].

#### 4.2.2. Inkjet 3D Printing

**Figure 4:**
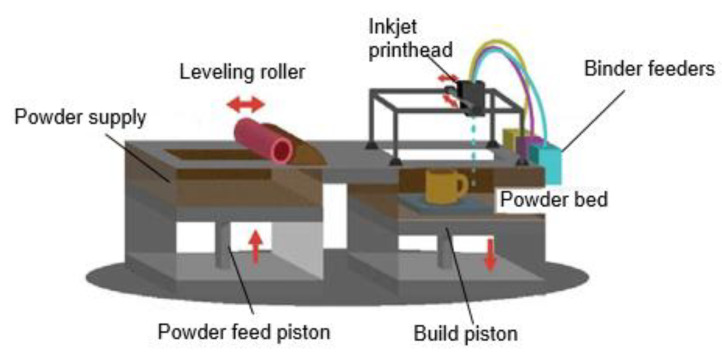
Schematic of the binder jetting process, a form of inkjet 3D printing (Adapted with permission from Ref. [140] Copyright 2016, Global Society of Scientific Research and Researchers).

Inkjet 3D printing involves the deposition of liquid drops with diameters in the micrometer range onto a substrate at a user-defined location after being expelled by either acoustic or thermal forces. The binder jetting process is one of the AM techniques whereby the inkjet head (IJH) technology is used for processing materials, as can be seen in Figure 4. Nanocellulose-based inkjet inks have attracted a lot of attention lately [132,133].

#### 4.2.3. 3D Spinning

**Figure 5:**
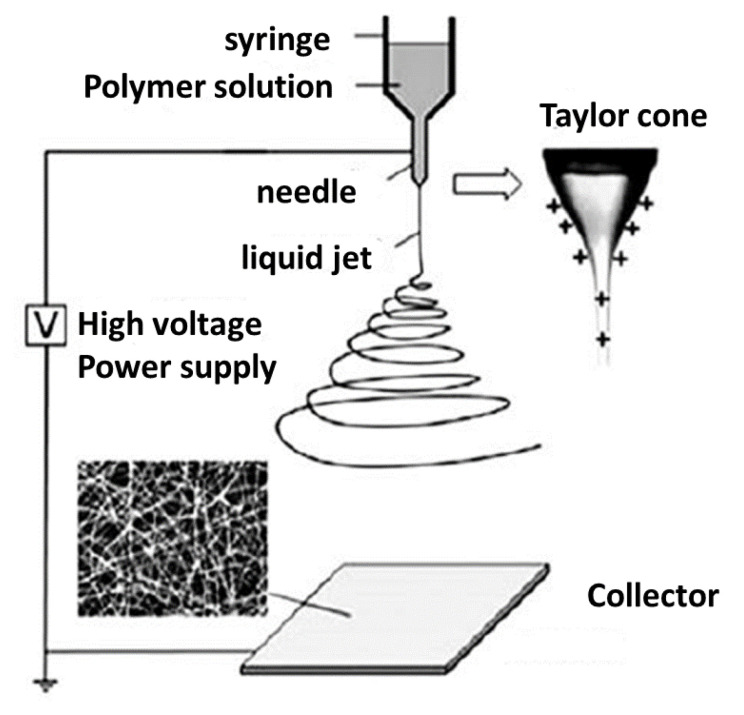
Schematic of the electrospinning technique (Adapted with permission from Ref. [141] Copyright MDPI, Basel, Switzerland).

Renewable sub-micron fibers and ultra-fine filaments with a robust, orientated structure have been produced using electro-, dry-, and wet-spinning of cellulose materials [137]. As reviewed by Atila et al. [138], using cellulose solution or derivatives, porous scaffolds with high resolution have been created using 3D electro-, dry-, or wet-spinning. To deal with electrical instabilities, techniques for precise deposition were used. According to Lee and Kim [139], a controlled sequential stacking of sub-micron filaments creates a three-dimensional scaffold structure. The experimental setup for the 3D electrospinning technique is shown in Figure 5.

### 4.3. Polymers Reinforced with Biobased Fillers in AM

Lignocellulose powder, a biobased material, can be utilized in AM as a filler material in combination with conventional polymers. Wood powder has been used as a filler in AM and has shown some potential application in printed parts. Rosenthal et al. [142] and Kariz et al. [143] implemented 3D printing of wood flour using binders and adhesives in the mixtures. Their findings demonstrated the viability of lignocellulosic material use in 3D printing procedures. The 3D printing of wood-based bulk materials, however, was said to demonstrate poor mechanical properties and thus unsuitable for structural applications.

In order to deal with the issue of poor mechanical properties, a variety of higher-quality polymer filaments containing lignocellulose have been developed. Le Duigou et al. [144] demonstrated the FDM of wood fiber-reinforced biocomposites by using a commercial filament known as fine woodfill filament. This filament was a combination of poly (hydroxyalkanoate) (PHA) and poly (lactic acid) (PLA) matrix reinforced with recycled wood fibers (10–20 wt. %). Their study revealed that mechanical characteristics were highly influenced by the printing orientation (0 or 90°) that was caused by fiber anisotropy. The porosity of printed items was altered by the printing width (100, 200, and 300%), thus impacting their mechanical characteristics. Other observations from the printing of the biocomposites included high porosities (approximately 20%), water absorption and swelling.

Tao et al. [124] developed PLA/wood flour (5%) biocomposite filament for FDM. The paper reported that the PLA fracture surface’s microstructure was altered by the addition of wood flour, thus improving the composite’s initial deformation resistance. Further, the composite’s initial thermal degradation temperature was reduced slightly. The research could be developed further by enhancing the interface compatibility between PLA and wood flour by improving the wood flour ratio.

By using a single-screw compound extruder, biofilaments made of micronized cocoa shell waste (CSW) and biodegradable polymer poly (ε-caprolactone) PCL were developed and characterized by Tran et al. [126]. They investigated surface and cross-section microstructure analysis using SEM, an Attenuated Total Reflectance (ATR) accessory coupled to a Fourier Transform Infrared (FTIR) Spectroscopy was used to analyze the biofilaments’ chemical composition and intermolecular interactions. To assess the biofilaments’ level of crystallinity at room temperature, X-ray diffraction (XRD) spectroscopy was used. Using an Instron dual-column tabletop universal testing system, the tensile strength of filaments was examined. Thermogravimetric analysis (TGA) was used to analyze the biofilament’s thermal stability (TGA). Printability was tested using FDM on the Prusa i3.

**Figure 6:**
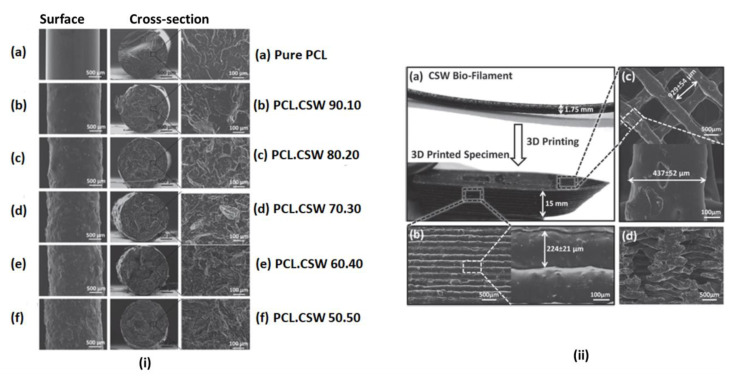
(**i**) (**a**) SEM images of the surface of pure PCL filaments revealed a morphology that was smooth, compact, and nonporous. The cross-section of the pure PCL revealed internal lamellae-like patterns (**b**–**f**) shows how the exterior surface and cross-section morphology of the biofilaments become coarser as the amount of micronized CSW increases (from 10% to 50% wt.). However, cross sections from all the CSW-containing biofilaments showed that the CSW particles were evenly distributed throughout the PCL matrix and were dense and homogenous. (**ii**) (**a**) Photographs of PCL/CSW (80.20) biofilament and resultant 3D printed specimen (**b**) SEM images of outer surface shows layers of deposited PCL/CSW struts that are well spaced, well-connected and have a thickness of 224 ± 21µm, showing good printing resolution., (**c**) SEM images of the inner structure reveals the crisscross pattern that forms the object’s horizontal architecture was likewise printed quite consistently, and (**d**) vertical cross section of inner structure of the 3D printed specimen showed a layered crisscross structure depicting a highly repeatable and compact printing technique (Adapted from Ref. [126]) (Copyright 2017 WILEY-VCH Verlag GmbH & Co. KGaA, Weinheim).

Figure 6i shows the microstructure of the developed biofilaments from pure PCL to up to 50% wt. of micronized cocoa shell waste content. Pure PCL showed a smooth, non-porous and compact external surface morphology with lamellae-like features in the internal surface cross-section. Increasing the amount of CSW from 10–50 wt. %. indicates a transformation into coarser internal and external structures. However, cross-sections from all the CSW-containing biofilaments showed that the CSW particles were evenly distributed throughout the PCL matrix and were dense and homogenous. It was discovered that adding CSW to PCL had no significant impact on the resultant biofilaments’ Young’s modulus. Pure PCL had 304 ± 20 MPa, and the biofilament with 30 wt. % CSW had the highest, at 356 ± 9 MPa. The amount, dispersion and interactions of the fillers and matrix have been reported to influence the mechanical properties of compounded polymers produced by extrusion processes [145].

Pure PCL, PCL.CSW 90.10, 80.20 and 70.30 filaments were tested for printability using FDM 3D printing. The higher percentages of 40 wt. % and 50 wt. % experienced printing challenges due to nozzle clogging of the 3D printer.

The outer surface of the specimen, which had uniform layers of deposited PCL/CSW struts, is shown in Figure 6ii, illustrating good printing resolution. The lack of gaps and distorted areas between layers served as a visual indicator of printing process uniformity. All these indications confirmed the feasibility of printing polymers (PCL) with the addition of fillers (CSW) for applications such as biomedical and household items.

Tarres et al. [146] produced biocomposites from fibers made of thermo-mechanical pulp (TMP) and two biobased polyethylenes (BioPE). Maleic anhydride-functionalized polyethylene (MAPE) was utilized as a coupling agent to increase the compatibility of the fibers with the BioPE. The effect of polyethylene functionalized with MAPE on the mechanical characteristics was measured. A maximum improvement in tensile strength between 115 and 127% was realized for biocomposites containing 6% *w*/*w* of MAPE and 30% *w*/*w* TMP fibers as compared to neat BioPEs. Due to the challenges of printer nozzle clogging, filaments comprising 10% and 20% TMP fibers were produced using extrusion and utilized in FDM.

**Figure 7:**
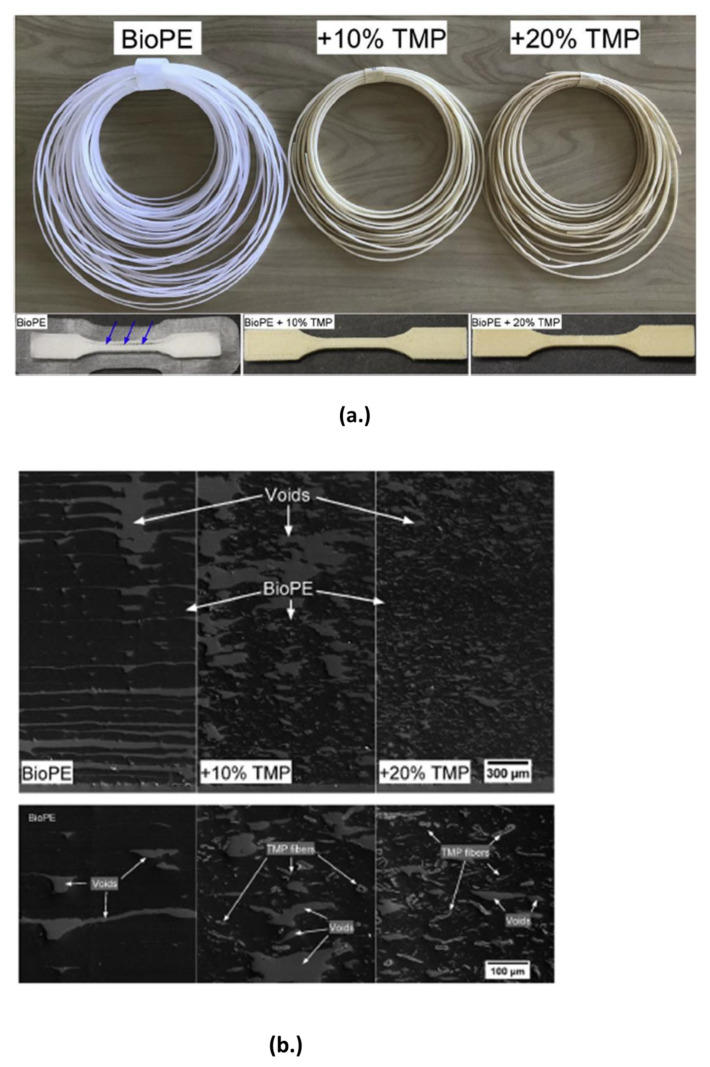
(**a**) Filaments and printed specimen of neat BioPE, 10% and 20% of TMP. (**b**) SEM images of the printed samples from BioPE and its biocomposites (Adapted from Ref. [146]) (Copyright 2018 Elsevier Ltd.).

Figure 7a illustrates the filaments manufactured and the printed tensile specimen. The arrows depict the presence of cracks that resulted from the printed layers’ weak lateral adhesion. This was not seen in the case of the biocomposites containing the TMP fibers, an indication that there was an improvement in the printability of the developed biocomposites. From Figure 7b presence of large voids in the BioPE sample could be observed from the SEM images, an indication of weak adhesion between printed layers. These voids were seen to reduce in size in the biocomposites, an indication of improved adhesion between the printed layers. Though the researchers demonstrated an improvement in the printability and mechanical strength of the biocomposites with TMP fillers, more research is required to explain these enhancements.

**Figure 8:**
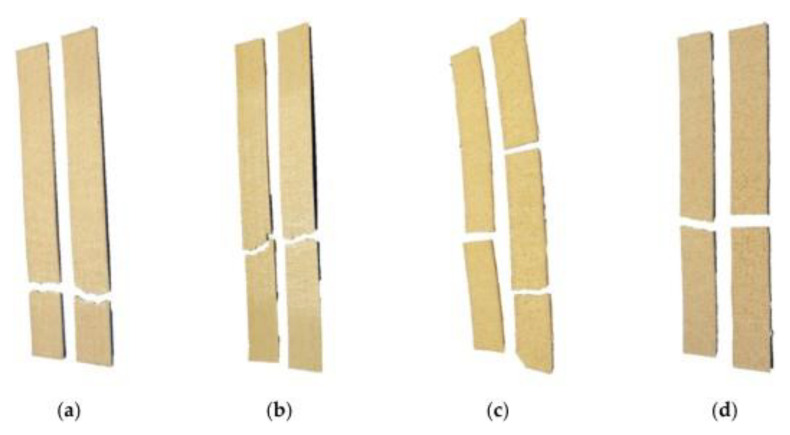
Fractured tensile test specimen: (**a**) rPP/RH 5 wt.% at 0°; (**b**) rPP/RH 10 wt.% at 0°; (**c**) rPP/RH 5 wt.% at 90°; (**d**) rPP/RH 10 wt.% at 90° (Adapted with permission from Ref. [7] (Copyright 2021 by the authors. Licensee MDPI, Basel, Switzerland.).

Wang et al. [125] developed a methodology for the preparation of 3D printable biocomposite filaments from micro/nanocellulose and polylactic acid (MNC/PLA). The schematic is shown in Figure 8. They demonstrated that the use of a silane coupling agent (KH-550) enhanced the interfacial compatibility of MNC and PLA. The best condition for the biocomposites was found to be 30 wt. % MNC modified with KH550, 5 wt. % PEG6000 (a plasticizer), and 65 wt. % PLA. With a tensile strength of 59.7 MPa, an elongation at break of 12% and flexural strength of 50.7 MPa, the mechanical properties of printed parts made from the MNC/PLA composites were maintained at a level comparable to that of neat PLA. The researchers successfully demonstrated the use of biocomposites in 3D printing where desirable characteristics such as high mechanical strength, lightweight, biodegradable and waterproof could be achieved for functional applications.

Morales et al. [7] synthesized and characterized biocomposite filaments from recycled polypropylene (PP) and rice husks (RH), a byproduct of agriculture. The fiber content ratio considered was 0, 5 and 10 wt. %, which was hand mixed before feeding into the extruder for filament fabrication. The researchers investigated how the fiber content ratio affected the physical, morphological, mechanical and thermal characteristics of printed objects using the Fused Filament Fabrication process. Due to the presence of lignocellulosic fiber components, thermo- gravimetric measurements showed that the degradation process for the biocomposites began much earlier than it did for the neat rPP.

The analysis of mechanical properties revealed that specimens printed with a raster angle of 0° had greater tensile strength than specimens printed at 90°, resulting due to poorer inter-layer bonding compared to in-layer bonding. These results are shown in Table 7. In other words, a lack of chemical bonding between the RH and rPP and their different surface energies existed, causing low dispersion of RH on the rPP matrix. The net effect was the reduced tensile strength of the biocomposites compared to neat rPP. Images from the scanning electron microscope (SEM) also revealed that there was little interaction between the untreated fiber and polymer matrix, which resulted in decreased tensile strength. Biocomposites, however, showed less warping than neat rPP during the printing process.

Figure 8 shows the test specimen at different failure modes according to ASTM D3039. The biocomposite with 5 wt. % of RH at a raster angle of 0° failed to angle at the bottom grip. This failure mode was denoted as the angled gauge bottom (AGB) failure mode. The biocomposite with 10 wt. % of RH at 0° failed to angle in the middle. This was denoted as angled gage middle (AGM) failure mode. The biocomposites with a raster angle of 90° failed laterally at the middle gage denoted lateral gage middle (LGM). The failure occurred within the bonded layers, in the direction of layer deposition, more brittle and in the gauge region for the specimens with a 90°raster angle. Due to the hydrophilic characteristic of natural fibers, rPP/RH biocomposites absorbed more water and swelled to a larger diameter than the neat PP.

This study concluded that, even though the mechanical characteristics of the polymer were greatly reduced when RH was added, they were still desirable for a variety of applications such as light-weight custom jigs and fixtures, concept pieces and thermoformed parts. The research, however, did not select the best ratio for the fiber content. The researchers only noted that the difference between the 5 and 10 wt. % was insignificant, making rice husks a good natural filler for polymers.

## 5. Current Challenges and Future Directions

Despite the impressive advancements reported recently in additive manufacturing technologies that have expanded in different types of material exploration, the feedstock advancements are, without a doubt, a crucial foundation for the large-scale application of these technologies in many areas. To improve biopolymers’ material flaws and qualities, such as printability, mechanical characteristics, rates of degradation and biocompatibility, challenges in 3D printing of bioplastic must be overcome [147].

One of the main challenges of using fiber-reinforced bioplastics is ensuring the uniform distribution of fiber in the polymer’s matrix and adequate stress transmission between fibers and matrix. These issues can be solved by modification of the fiber surface and the matrix as well as by developing a suitable processing technique. The weakest point of a bioplastic will reduce its strength. Therefore, caution must be taken to avoid weakening or damaging the reinforcing fibers during modification and processing, which may occur during thermoplastic processes such as melt-blending process, extrusion pro cess and pelletizing [15,147].

It has been anticipated that nanocellulose (a common reinforcement for biocomposites) with an appropriate degree of nanofibrillation, a suitable surface chemistry and an appropriate surface morphology would be advantageous for enhancing the mechanical properties of a biocomposite material. However, it has been difficult to fully utilize nanocellulose as a reinforcing element in biocomposites primarily because of difficulties like dewatering or drying without causing agglomeration of the nanomaterials and the ensuing production costs. As a result, the material may not disperse properly in the polymer matrix, thus limiting the material’s capacity as a reinforcement [148,149].

In order to achieve adequate component interaction and the appropriate biocomposite properties, uniform dispersion of fibers or particles in the polymer matrix is necessary for biocomposites by avoiding agglomeration caused by fibers interaction, entanglement and the incompatibility of hydrophilic fibers with hydrophobic matrixes [150]. One typical method for dispersing fillers in a bioplastic matrix is to first combine dry polymer and compatibilizer powder before adding the fillers. To functionalize the fillers, chemical or mechanical pretreatment techniques may be used. It may be necessary to dry natural fillers before processing them because they are hydrophilic. The biocomposite powder that has been dried and blended can either be put straight into a melt extruder or can first be melt compounded, pressed and chopped into pellets. The mixture will be further compounded by melting and mechanical shearing inside the extruder. Pelletizing and melt extrusion are stages that can be done numerous times, but it should be remembered that each time this is done, fiber damage happens [151,152]. The extrusion process parameters can be studied and optimized to achieve the desired property of the developed bioplastic.

Another challenge with AM of bioplastics is the poor interaction between the fillers and the polymer matrix due to the difference in polarities. The fillers are hydrophilic in nature, whereas the polymers are hydrophobic in nature. Therefore, it’s crucial to use coupling agents and/or fiber sizing, which balances out the polarity difference between the filler and matrix to increase the strength of the adhesion between the two phases and aid in distributing the fibers throughout the matrix [15]. Fillers can be physically altered to alter their surface characteristics and promote mechanical adhesion or chemically altered to enhance adhesion through chemical reactions. Ultraviolet (UV), plasma, and corona are some of the physical treatments [153], whereas alkaline, oxidation, silane [154], grafting [155], acetylation, and maleated coupling agents [156] are some of the chemical treatments [157].

Maleated coupling agents, such as maleated polypropylene (MAPP) or maleated polyethylene (MAPE), are frequently employed for lignocellulosic fibers and thermoplastic polymers [158,159]. Maleated polyolefin with the PE portion made from biomass resources has also been developed recently [160]. In biocomposites, the polymeric matrix typically contains 10–50 wt. % fibers and 0–8 wt. % coupling agents [161,162].

Biodegradable bioplastics have been discovered to be useful in the biomedical field, including the manufacture of bone plates and screws, carriers for drug delivery and scaffolds in tissue engineering [163,164]. To test for the biodegradability of the bioplastic, it is advisable that the methodology be chosen based on the polymer’s probable application and its end-of-life which are compost, soil or aquatic systems. Though there are a number of standards available to analyze the rate of degradation of bioplastics, they don’t give comparative results. It is important that comparable international standards for biodegradability be developed. An important research area could be how the biodegradability of biopolymers is tuned, especially for the biomedical industry.

Characterization techniques that are designed for the intended application can be instrumental in determining the strengths and weaknesses of printed parts from biocomposites. The most important properties in 3D printed parts from biocomposites include tensile strength, Izod impact resistance, flexural properties, thermal properties and morphological properties [165]. As for the 3D printing process, modification of the commercial printers to accommodate the natural fillers in biocomposites, optimization of printing parameters as well as the development of various post-processing technologies will help improve the printability of developed biocomposites from natural fillers.

Biocomposites that are biocompatible can be utilized to develop stimuli-responsive materials (SRMs) such as shape memory polymers (SMPs). This is a recent development in the biomedical field where four-dimensional (4D) bioprinting is gaining ground. The 3D-printed tissues undergo remodeling and maturation during 4D bioprinting. The main goal of the 4D bioprinting (4DBP) technology is to create complex tissue constructs that have the ability to change their shape and characteristics in response to various physical, chemical, or biological stimuli and aid in the growth, repair or replacement of tissues, cells or organs that have been damaged [166]. This emerging technology creates a potential research area for biocompatible biocomposites to be used in tissue engineering applications.

## 6. Conclusions

Advancements in the 3D printing of bioplastics present a viable production strategy by lowering waste production and energy consumption. The use of bioplastics in 3D printing has been studied as a potential solution to the problems posed by petroleum-based materials, such as the scarcity of resources and unfavorable environmental effects. To improve the quality of 3D-printed products made from bioplastics and expand their applications, numerous studies in printing technology, printing feedstock, properties, and applications have to be carried out. Some of the biobased materials that have been used in 3D printing include starch, lignin, cellulose and whole biomass. The commonly used AM technologies for printing biocomposites are fused deposition modeling, direct ink writing, micro-extrusion 3D bioprinting, inkjet 3D printing and 3D spinning. These technologies enable the production of composite materials for the biomedical, pharmaceutical, food and construction industries using inexpensive materials produced from biomass.

The relationship between material structure and the printing process is crucial in determining the property of the final product. The intermolecular structure of the bioplastic material, such as crystallinity, material anisotropy and interfacial interactions, must therefore be well understood in order to achieve the desired properties of the 3D printed part. To make 3D printing from bioplastics competitive with traditional material fabrication technologies, further engineering is required for a number of printing parameters, including the printing temperature, resolution, layer thickness and component production rate.

Utilizing compatibilizers and altering the interfacial chemistry may improve the distribution and bonding of plastics with fillers made from biomass. This could greatly increase the concentration of fillers with moderate strength and help to at least partially prevent the depletion of petroleum-based materials.

## Figures and Tables

**Table 1:** Bioplastics composition, properties and applications.

No.	Bioplastic Composition	Properties	Application	Ref.
1	Poly(lactic acid) and poly (butylene adipate terephthalate)	- Dense matrix - Enhanced crystallinity - Reduced permeation to oxygen and moisture	- Bakery packaging - Seafood packaging - Active packaging	[39]
2	Chitosan, polyethylene glycol and methyl ether methacrylate	- Antioxidant - Antimicrobial nature - Enhanced mechanical properties	- Active food packaging	[40]
3	Thermoplastic starch	- Improved polymer compatibility - Reduced tensile strength - Antimicrobial nature	- Fresh noodles	[41]
4	Cassava starch	- Optimized tensile strength and elastic modulus	- Packaging	[42]
5	Soya protein	- Super absorbent - water absorption capacity	- Zinc release in soil	[43]
6	Poly vinyl alcohol/poly (lactic acid)	- Higher encapsulation, efficiency and stability	- Controlled release of fertilizers	[44]
7	Gelatinized starch	- Higher swelling degree with delayed release of nutrients	- Sustained release of micronutrients	[45]
8	Seaweed extract and calcium alginate	- pH-responsive water holding capacity	- Improved soil hydro physical and fertility	[46]
9	Poly vinyl alcohol/poly (lactic acid)	- Compressibility - Mineralization behavior - Biocompatibility of the viscoelastic characteristic to natural cartilage	- Tissue engineering	[47]
10	Chitosan and biguanidine	- Sequential release behavior of the vascular endothelial	- Injectable - Bone tissue engineering	[48]
11	Starch/hydroxyapatite	- Low cost - Wide availability - biocompatibility	- Biomedical device	[49]
12	Rice starch extract coated with iron oxide nanoparticles	- Biological active - Photo acoustic imaging	- Chemo—photo thermal therapy	[50]

**Table 3:** Methodologies to assess biodegradation in compost.

Biopolymer	Origin of Compost	Methodology	Ref.
PLA, PLA/Nanocellulose (Gum)	Food waste	ASTM D5338, chemical/morphology analysis, mass loss, thermal/microorganism characterization	[74]
PVA, PVA/Nanocellulose (CNF)	MSW	ASTM D5338, mass loss, morphology, visual and chemical analysis	[75]
PHA-based	MSW	ASTM D5338 and ISO 20200	[81]
PVA/Starch	MSW	ISO 14855-1, ISO 20200, thermal/microorganism characterization	[82]
PLA, PLA/TAC and PLA/PHB/TAC	MSW	CO_2_ concentration, mass loss, chemical analysis, thermal characterization	[83]
Nano-reinforced PLA	MSW	ISO 16929, mass loss and microorganism characterization	[84]
PLA and PLA/Silica	Compost fermented with biomass	Modified ISO 17556	[85]

**Table 4:** Methodologies to assess biodegradation in aquatic systems.

Standard	Approach
ISO 19679:2020	“Plastics—Determination of the aerobic biodegradation of non-floating plastic items at the seawater/sediment interface—Analyses amount of evolved CO_2_”
ISO 18830:2016	“Plastics—Determination of the aerobic biodegradation of non-floating plastic items at the seawater/sediment interface—Measures the demand for O_2_ in a closed respirometer”
ISO 14853:2016	“Plastics—Determination of the ultimate anaerobic biodegradation of plastic items in an aqueous system—Technique by measurement of biogas generation”
ASTM D7991-15	“Standard Test Procedure for Measuring Aerobic Biodegradation of Plastics Buried in Sandy Marine Sediment in a Controlled Laboratory”
ASTM D7473-12	“Standard Test Procedure for Weight Attrition of Plastic Materials in the Marine Environment by Open System Aquarium Incubations”
ASTM D6691-17	“Standard Test Procedure for Determining Aerobic Biodegradation of Plastic Materials in the Marine Environment by a Natural Sea Water Inoculum or Defined Microbial Consortium”

**Table 5:** Different properties of the main types of nanocellulose [103].

Properties	NFC	CNC	BNC
Size	Diameter (1–10 µm)	Diameter (5–30 nm)	Diameter (20–100 nm)
Aspect Ratio	Very high	Low	Low
Reagent type	Corrosive	Corrosive	Non-corrosive
Sustainability	Not very sustainable	Not very sustainable	Green approach
Cost	Low cost	Low cost	High cost
Energy	High energy process	High energy process	Green process

**Table 6:** Cellulose-reinforced plastic filaments for FFF.

Biocomposite	Filament Fabrication	FFF Printer	Printing Temp. (°C)	Potential Application	Ref.
PLA/wood flour 5%	Single-screw extruder	Self-assembled FDM 3D printer	210, 0.4 mm nozzle	Functional load-bearing application	[124]
Micro/nanocellulose polylactic acid (MNC/PLA) composite (30 wt.% MNC + 5 wt.% PEG6000 + 65 wt.% PLA)	Twin -screw extruder	FDM Desktop 3D printer (Z603S)	190, 0.4 mm nozzle	Structural applications	[125]
PCL/Cocoa Shell Waste (0–50%)	Single -screw extruder	Prusa i3	120, 0.3 mm nozzle	Household and biomedical application	[126]
Thermoplastic copolyester (TPC)/Soybean Hull Fiber (5–10%)	Capillary rheometer	Desktop FFF machine (Printrbot)	220, 0.5 mm nozzle	Functional application	[27]
PLA/cotton cellulose (0–20%)	2-step extruder	Lulzbot TAZ 5 3D printer	210	Automotive industry	[127]

**Table 7:** Mechanical properties of rPP and rPP/RH biocomposites at different printing raster angles.

Raster Angle	Specimen	Tensile Strength (MPa)	Tensile Elongation (%)	Young Modulus (GPa)
0°	rPP	26.02 *±* 0.47	6.16 *±* 0.19	1.34 *±* 0.05
	rPP/RH (5 wt.%)	13.62 *±* 2.71	4.10 *±* 0.20	1.06 *±* 0.13
	rPP/RH (10 wt.%)	13.78 *±* 0.59	5.06 *±* 0.16	1.04 *±* 0.04
90°	rPP	4.33 *±* 1.73	1.01 *±* 0.35	0.74 *±* 0.37
	rPP/RH (5 wt.%)	7.92 *±* 0.67	2.04 *±* 0.43	1.01 *±* 0.12
	rPP/RH (10 wt.%)	5.66 *±* 0.82	3.07 *±* 0.45	0.66 *±* 0.13

## Data Availability

Data sharing does not apply to this article as no datasets were generated or analyzed during the current study.
